# Molecular Details on Multiple Cofactor Containing Redox Metalloproteins Revealed by Infrared and Resonance Raman Spectroscopies

**DOI:** 10.3390/molecules26164852

**Published:** 2021-08-11

**Authors:** Célia M. Silveira, Lidia Zuccarello, Catarina Barbosa, Giorgio Caserta, Ingo Zebger, Peter Hildebrandt, Smilja Todorovic

**Affiliations:** 1 Instituto de Tecnologia Química e Biológica António Xavier, Universidade NOVA de Lisboa, Av. da República, 2780-157 Oeiras, Portugal; celiasilveira@itqb.unl.pt (C.M.S.); lidiazuccarello@itqb.unl.pt (L.Z.); catarina.barbosa@itqb.unl.pt (C.B.); 2 Institut fur Chemie, Sekr. PC14, Technische Universitat Berlin, Strasse des 17. Juni 135, D-10623 Berlin, Germany; giorgio.caserta@tu-berlin.de (G.C.); ingo.zebger@tu-berlin.de (I.Z.); peter.hildebrandt@tu-berlin.de (P.H.)

**Keywords:** vibrational spectroscopy, metalloproteins, resonance Raman spectroscopy, IR, heme proteins, Fe-S clusters, hydrogenases

## Abstract

Vibrational spectroscopy and in particular, resonance Raman (RR) spectroscopy, can provide molecular details on metalloproteins containing multiple cofactors, which are often challenging for other spectroscopies. Due to distinct spectroscopic fingerprints, RR spectroscopy has a unique capacity to monitor simultaneously and independently different metal cofactors that can have particular roles in metalloproteins. These include e.g., (i) different types of hemes, for instance hemes *c*, *a* and *a_3_* in *caa_3_*-type oxygen reductases, (ii) distinct spin populations, such as electron transfer (ET) low-spin (LS) and catalytic high-spin (HS) hemes in nitrite reductases, (iii) different types of Fe-S clusters, such as 3Fe-4S and 4Fe-4S centers in di-cluster ferredoxins, and (iv) bi-metallic center and ET Fe-S clusters in hydrogenases. IR spectroscopy can provide unmatched molecular details on specific enzymes like hydrogenases that possess catalytic centers coordinated by CO and CN^−^ ligands, which exhibit spectrally well separated IR bands. This article reviews the work on metalloproteins for which vibrational spectroscopy has ensured advances in understanding structural and mechanistic properties, including multiple heme-containing proteins, such as nitrite reductases that house a notable total of 28 hemes in a functional unit, respiratory chain complexes, and hydrogenases that carry out the most fundamental functions in cells.

## 1. Introduction

Vibrational spectroscopy has, over the past couple of decades, provided valuable information on structure-function relationship in proteins, contributing to the elucidation of molecular mechanisms, dynamics and interactions on the level that goes beyond high-resolution crystallographic structures. It does not depend on size or paramagnetic properties of the protein like nuclear magnetic resonance (NMR) and electron paramagnetic resonance (EPR) spectroscopies, and furthermore, it can be coupled with electrochemical methods and employed in time-resolved mode, offering the possibility to probe redox and transient molecular events down to the femtosecond time scale [1].

**Figure 1:**
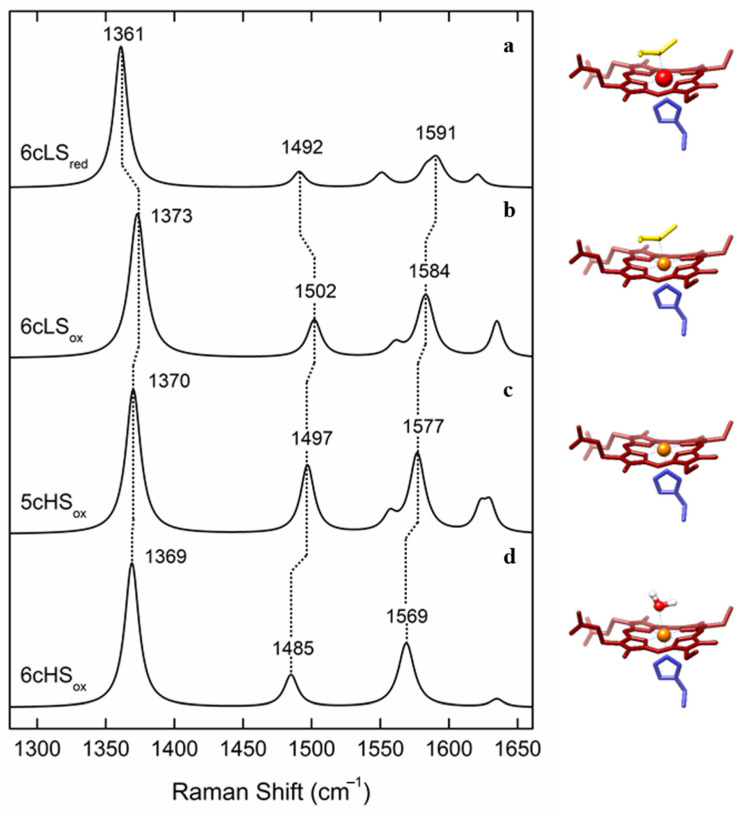
Simulated resonance Raman (RR) spectra of different oxidation, spin, and coordination states of cytochrome *c*, with depicted marker bands, left, and corresponding heme configurations, right. (**a**) Ferrous (Fe^2+^) heme in low-spin (LS) state, coordinated by distal Met and proximal His (6cLS). (**b**) Ferric (Fe^3+^) heme in 6cLS state, coordinated by distal Met and proximal His. (**c**) Ferric heme in a high-spin (HS) state, coordinated by proximal His and vacant 6th axial position (5cHS). (**d**) Ferric heme in 6cHS state, coordinated by a distal water molecule and proximal His.

In the case of metalloproteins, the intrinsically low sensitivity and the selectivity of Raman spectroscopy are strongly increased when the energy of the incident laser light is in resonance with an electronic transition of the chromophore. The resultant resonance Raman (RR) spectra display several orders of magnitude higher intensity for the vibrational modes of the cofactor, regardless of the size of the protein matrix [1]. RR spectroscopic studies have mainly focused onto metalloproteins containing iron and copper ions, but they have also been reported for other transition metals such as nickel, cobalt, and molybdenum [2,3,4,5]. The studies of heme and non-heme iron, iron-sulfur cluster and copper-containing metalloproteins have provided a wealth of details on their active sites, hence contributing to understanding of their mechanistic properties. In particular, RR spectra of heme proteins obtained upon Soret-band excitation are, in the high-frequency (1300–1700 cm^−1^) region, dominated by the core-size marker bands *ν*_i_ (*ν*_4_, *ν*_3_, *ν*_2_ and *ν*_10_) originating from complex porphyrin vibrations that are sensitive to the redox, spin, and coordination state of the heme iron (Figure 1).

For instance, a five coordinated high-spin (5cHS) heme has an increased size of the iron ion in comparison with six-coordinated low-spin LS (6cLS) heme, which is reflected by a frequency downshift of the corresponding *ν*_3_ and *ν*_2_ modes (Figure 1, Table 1) [1]. The spectral distinction between LS and HS heme populations is therefore straightforward in RR spectra, which has helped addressing, e.g., processes at catalytic HS vs. electron transfer (ET) LS hemes in oxygen and nitrite reductases and the activation mechanism of cytochrome *c* peroxidases (C*c*Ps) [6,7,8,9]. Similarly, reduction of the heme iron, while maintaining the spin and coordination state, causes an increased back-donation of electron density into the π* orbital of the ferrous heme porphyrin and thus results in a downshift of the *ν*_4_ mode frequency (Figure 1, Table 1) [1]. The capacity of RR spectroscopy to distinguish between ferric and ferrous heme populations has contributed to disentangling of the ET processes in numerous metalloproteins, as well as those that involve semi-reduced forms of Complex III and dihemic bacterial C*c*Ps [9,10].

RR spectra of Fe-S cluster-containing proteins, obtained with a laser wavelength that matches the energy of S → Fe charge transfer (CT) transitions, show selectively enhanced modes involving the metal–ligand stretching coordinates in the low-frequency (200–450 cm^−1^) region (Table 1). The spectra are sensitive to the cluster type, structure, and symmetry and moreover allow for distinguishing between bridging vs. terminal Fe-S vibrational modes that predominantly involve inorganic and cysteine-bound S atoms in each cluster, respectively [11]. Unlike in the case of heme proteins, in Fe-S proteins usually only one redox state is RR active; [2Fe-2S]^2+^, [3Fe-4S]^1+^, and [4Fe-4S]^2+^ (and [4Fe-4S]^3+^ in HiPIPs). RR spectroscopy can simultaneously probe different types of clusters present in the same protein, alongside with processes that account for their interconversion [11].

Large enhancements of Raman signals are obtained by measuring the spectra of molecules located in close proximity to plasmonic metals (e.g., Ag and Au), due to the surface enhanced Raman (SER) scattering effect [1]. Accordingly, SER spectroscopy can provide sensitive (bio)chemical information about the studied system, representing a promising analytical tool for, e.g., medical diagnostics [12,13,14]. The combination of the SER and RR effects (SERR spectroscopy) enables sensitive and selective probing of the chromophoric part of molecules, such as the cofactors of metalloproteins attached to SER active metal surfaces [1]. SERR spectroscopy can provide specific molecular information about proteins that perform their physiological function in the immobilized state (e.g., membrane proteins). In the case of heme proteins, the conditions for SERR signal enhancement are met upon immobilization on plasmonic Ag structures, for which the surface plasmon energy matches that of the heme Soret electronic transition, when using a laser excitation line around 410 nm. Thus, SERR spectra of heme proteins reveal the oxidation, spin, and coordination states of the heme group, but exclusively of molecules immobilized on plasmonic surfaces. In addition, the nanostructured metal that amplifies the signals can serve as a working electrode, allowing for spectro-electrochemical SERR studies capable of monitoring the changes of the heme group as a consequence of variations of the electrode potential [1,15]. This approach can reveal redox potentials (*E*^0^) of distinct and simultaneously present heme groups in immobilized proteins, e.g., *E*^0^ (HS) vs. *E*^0^ (LS), which is particularly relevant for understanding of ET pathway in membrane proteins [6,16,17].

**Figure 2:**
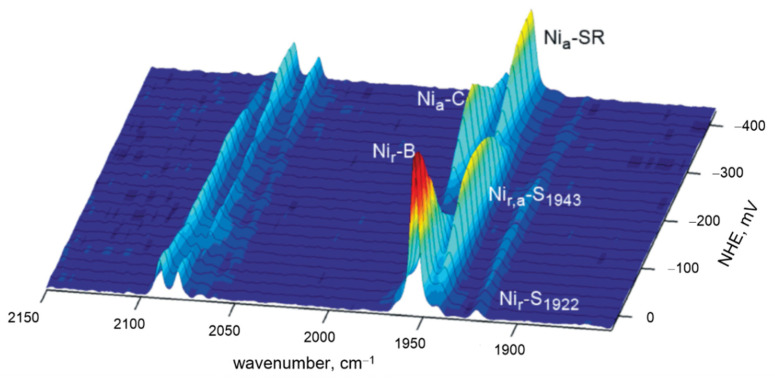
Infrared (IR) potentiometric titration of a [NiFe] hydrogenase in solution, shown in a three-dimensional representation. Each slice represents a spectrum recorded at a designated potential. Redox transition of the active site can be monitored via the characteristic IR absorptions of the CO and CN^−^ ligands between 1920 and 1965 cm^−1^ and 2050 and 2100 cm^−1^, respectively. Adapted from Millo et al. [19]. Copyright © (2009) American Chemical Society.

Infrared (IR) spectroscopy most commonly provides information on the secondary structure of proteins based on the analysis of the amide I (1600–1700 cm^−1^) and amide II (1480–1580 cm^−1^) bands [1]. However, when measured in the difference mode, IR spectra can probe structural changes of individual cofactors and sensitively detect redox-linked structural changes, protonation events or amino acids, and specific groups upon isotopic labeling (e.g., ^13^C and ^15^N labeled heme) [18]. Analogous to SERR, surface enhanced infrared absorption (SEIRA) spectroscopy takes advantage of protein molecules that are found in the vicinity of nanostructured metal surfaces deposited on a transmission window or an inert ATR (attenuated total reflection) crystal, experiencing an enhanced absorption of the incident IR radiation. SEIRA can be used to control the protein attachment and orientation at the biocompatible surface upon immobilization, but more importantly it can also provide fine details about redox-linked changes of the secondary structural elements when employed in spectro-electrochemical mode (*vide supra*) [1,15]. For certain metalloenzymes, where the active site harbors ligands that possess spectrally isolated IR absorption bands, such as hydrogenases, IR (transmission) spectroscopy and its surface sensitive variants offer unique insights in the redox behavior of the catalytic center (Figure 2) [19].

[NiFe] and [FeFe] hydrogenases, for example, contain bimetallic catalytic centers with unusual CO and CN^−^ ligands, exhibiting distinct IR bands related to the corresponding stretching vibrations that are normally centered between 1780 and 2150 cm^−1^ (Table 1). Their particular frequencies are sensitive to electron density distribution at the bimetallic center. While spectro-electrochemical studies in solution in the transmission mode probe individual redox transitions of the catalytic center and the steps of its activation, as displayed in Figure 2, surface sensitive ATR and SEIRA spectro-electrochemistry allows in situ studies under controlled gas atmospheres and potential control for a better understanding of the activation mechanism and catalysis [19,20,21,22].

Here, we review work on several multicofactor redox enzymes, which have been challenging for conventional, e.g., electronic absorption and EPR spectroscopies, and for which vibrational spectroscopy ensured true advancement in understanding of their structural and mechanistic properties. In the following, we (i) discuss multiple heme-containing proteins, such as nitrite reductases harboring an impressive total of 28 hemes in a functional unit, (ii) describe the work on Complex III and dihemic C*c*Ps, the heme groups of which can be distinguished via selective reduction due to large differences in the respective redox potentials, and (iii) dwell on oxygen reductases for which RR and SERR spectroscopy played a crucial role in disentangling the structure and formation kinetics of catalytic intermediates. In the last section, Fe-S-containing proteins, such as hydrogenases and di-cluster-containing ferredoxins, are depicted. The main focus is therefore given to the systems that carry multiple cofactors that can be simultaneously observed in RR spectra, for which in parallel IR spectroscopy can provide further details. The cases in which one of the cofactors has an intrinsically low absorption coefficient and is therefore not observed in (SE)RR spectra, such as the molybdopterin Moco cofactor of human sulfite oxidase that also houses a heme group, will not be discussed [15].

## 2. Heme Proteins

### 2.1. Nitrite Reductases (NiR)

**Figure 3:**
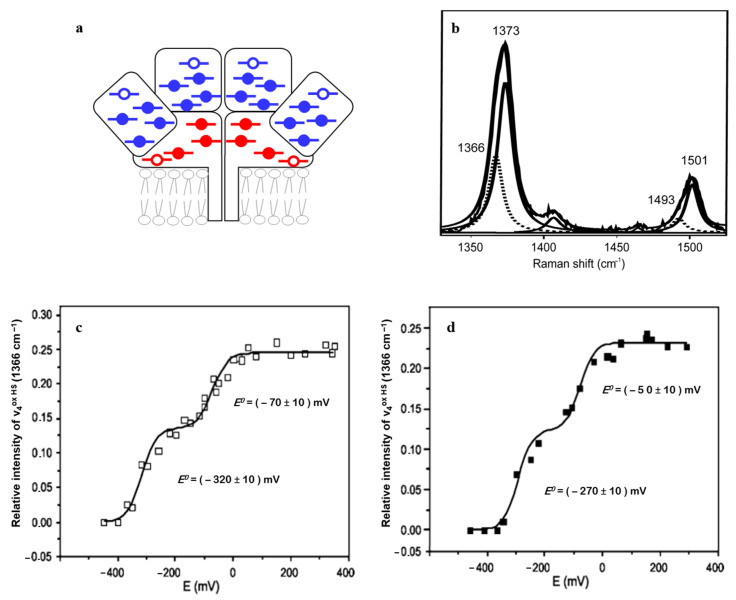
NrfHA nitrite reductase. (**a**) Schematic representation of 28 heme groups in a functional unit (with LS hemes depicted as solid and HS as empty symbols). (**b**) High-frequency RR spectrum of ferric NrfHA and its component analysis with designated *ν*_4_ and *ν*_3_ modes of the HS (dotted line) and LS (solid line) species. (**c**,**d**) Nernst plots based on variation of *ν*_4_ (HS) along SERR spectro-electrochemical titration of immobilized NrfHA in the (**c**) presence and (**d**) absence of 2-*n*-heptyl-4-hydroxyquinoline *N*-oxide, HQNO. Adapted from Todorovic et al. [6] and Martins et al. [23]. Copyright © (2010, 2012) American Chemical Society.

*NrfHA menaquinol: nitrite oxidoreductase* complex catalyzes the six-electron reduction of nitrite to ammonia in a reaction that involves eight protons. It houses a total of 28 heme groups in the biological unit and, as such, represents a challenge for every experimental approach. Twenty-two heme groups have 6cLS and six have 5cHS configuration (Figure 3a); two of the HS hemes are membrane-integrated and the other four represent catalytic sites that carry unusual Lys-coordination. The HS and LS hemes can be easily distinguished in the RR spectra of NrfHA, with redox-sensitive *ν*_4_ and redox/spin-sensitive *ν*_3_ modes of the HS species at 1366 and 1493 cm^−1^, and of the LS at 1373 and 1501 cm^−1^, respectively (Figure 3b), allowing for independent monitoring of the processes that involve these two populations. Binding of nitrite to the catalytic HS hemes and the resulting spin configuration of the initial enzyme/substrate complex have profound consequences for the reaction mechanism of nitrite reduction, indicating whether the N-O bond cleavage follows the homolytic or heterolytic route, and the subsequent steps of the catalytic cycle. RR data have provided the first experimental evidence that nitrite binding to NrfHA-active site HS hemes causes a spin conversion from HS to LS configuration, which implies that the heterolytic cleavage of the N-O bond is favored in the first step of the catalytic reaction [23].

SERR spectro-electrochemistry furthermore helped disentangle the ET pathway in NrfHA. Potentiometric titrations of NrfHA immobilized on biocompatible Ag electrodes was followed by the analysis of the ν_4_ band of the HS hemes only (1366 cm^−1^) in the presence/absence of 2-*n*-heptyl-4-hydroxyquinoline *N*-oxide (HQNO). The inhibitor selectively binds in the proximity of the membrane-integrated HS heme subpopulation (red empty symbols, Figure 3a). Out of the two redox transitions, observed at *E*^0^ = −270 ± 10 mV vs. NHE and at *E*^0^ = −50 ± 10 mV vs. NHE, only the former was modulated in the presence of HQNO (Figure 3c,d). The *E*^0^ which remained unaltered in the presence of the inhibitor was therefore assigned to the catalytic NrfA HS hemes. These SERR-based insights provided the first evidence for the downhill biological electron flow in the integral NrfHA [6].

*Cytochrome cd_1_ nitrite reductases* (*cd*_1_NiRs) catalyze the reduction of nitrite to NO as a part of bacterial dissimilatory denitrification pathway. They are homodimer proteins containing a *d*_1_-type heme in the active site and a *c*-type heme as ET center in each subunit [24,25]. Owing to their distinctive electronic absorption properties, the hemes *c* and *d*_1_ can be selectively probed by RR spectroscopy employing 413 (or 514 nm) and 457 nm laser excitation, respectively [26,27,28]. Upon binding of the reaction product, NO, the Soret band of heme *d*_1_ (ca. 460 nm) is blue shifted, allowing for both hemes to be probed with 413 nm excitation [28]. RR spectra of *cd*_1_NiR-NO adducts revealed that in addition to the heme *c*, two *d*_1_-heme spin configurations co-exist: a 6cLS-NO species and a 5cHS-NO state, in which the proximal His residue is detached from the heme [28]. The presence of the 5c adduct was confirmed by a 520 cm^−1^ mode whose frequency falls in the range of Fe-NO stretching modes characteristic for 5c-NO adducts of *c*-type cytochromes [29,30,31]. Conversely, the stretching coordinates of the 6cLS-NO *d*_1_ heme have been observed at unusually high frequencies (585 cm^−1^ in *Pseudomonas aeruginosa cd*_1_NiR) in comparison to other heme–NO complexes [29,30], which is thought to be related with the electronic properties and highly ruffled structure of heme *d*_1_ [27].

### 2.2. Heme-Containing Respiratory Chain and Analogous Complexes

*Complex III* (ubiquinol: cyt *c* oxidoreductase or *bc*_1_ complex) catalyzes the transfer of two electrons from ubiquinol to two cyt *c* molecules. *bc*_1_ complexes are formed by a minimum of three subunits, one contains a *c*_1_-type heme (or structurally and functionally analogous cytochrome *f* in plants, cyanobacteria, and green algae that house cytochrome *b_6_f* complex), one holds a Rieske type [2Fe-2S] cluster, and the third one contains two LS *b*-type hemes, designated *b*_L_ and *b*_H_ [10]. RR spectroscopy has been employed in the initial characterization of heme groups of the complex at different stages of reduction. Namely, due to the differences in midpoint redox potentials of the heme *c*_1_, low-potential heme *b* (*b*_L_) and high-potential heme b (*b*_H_), it is possible to selectively reduce the higher-potential sites (heme *c*_1_ and *b*_H_). A selective resonance enhancement using multiple excitation lines and sequential stoichiometric reduction of the complex allowed for spectral distinction between the *c* and *b*-type hemes, providing insights into the differences in peripheral heme–protein interactions, and in particular the conformation of vinyl substituents of the pyrrole rings [10].

RR spectra of cyt *c*_1_- and cyt *f*-containing subunits of *bc*_1_ and *b*_6_*f* complexes employing Q-band (550 nm) excitation effectively probe the respective local heme environments of these sites, indicating remarkably similar macrocycle geometry of the two hemes [32]. Cyt *b*_6_*f* participates in the oxygenic photosynthesis as a redox link between the two reaction center complexes. RR spectroscopy initially helped identify the chromophoric groups in *b*_6_*f* complexes isolated from different species, revealing a presence of chlorophyll *a*, *β*-carotene, and an additional 5cHS *c_1_*-type heme [33]. RR spectra of oxidized, native, ascorbate-and dithionite-reduced forms of spinach cyt *b*_6_*f*, obtained using 441, 413, and 406 nm lasers, reveal RR contributions of chlorophyll *a*, *β* carotene, the 5cHS *c*-type heme of cytochrome *f*, and the *b*-type hemes of cytochrome *b*_6_ of the complex [34]. RR bands arising from the pigments, found in 1520–1575 cm^−1^ range, are particularly intense in the spectra obtained with 441 nm [34]. Different conformations of the two *b*-type hemes, a strongly distorted vs. largely planar geometry, were observed and correlated to the differences in the redox potentials of the two hemes in cyt *b*_6_*f*, which appears to be a common feature for the *bc*_1_ and *b*_6_*f* complexes. A functional analogue to *bc*_1_ complex carrying two *b-*type hemes and one *a*-type heme was found in an archaeon. RR spectroscopy helped characterize the heme groups in this enzyme that show *bc*_1_ complex activity [35].

IR difference spectroscopic studies of the *bc*_1_ complex revealed redox-induced structural changes, while additional information obtained on site-directed mutants and site-directed labeling of cofactors ensured complete assignments of the observed vibrational modes [36]. More recently, ATR-IR spectroscopy was employed to study redox changes in thin layers of bovine *bc*_1_ complex that have been deposited on the surface of a silicon microprism. Thereby, redox-sensitive IR absorption bands in the potential-induced difference spectra of *c*_1_, *b*_H_, and *b*_L_ hemes, ubiquinone, and surrounding amino acid residues were identified upon selective reduction. A similar approach was used to probe the [2Fe–2S] cluster of the complex. The pH-dependent IR features in the reduced minus oxidized difference spectra revealed specific signals that were attributed to an imidazolate-to-imidazole transition, providing the first experimental evidence that a cluster coordinating His is the most likely candidate for redox-linked protonation site [37,38].

*Cytochrome c peroxidase*, C*c*P: Soluble, periplasmic, dihemic C*c*Ps catalyze the reduction of hydrogen peroxide to water by two electrons delivered from small redox proteins in some bacteria. These C*c*Ps contain two *c*-type hemes, one which is in analogy to Complex III, high-potential, H-heme (*E*^0^ = 330 to 450 mV vs. NHE), and the other low-potential, L-heme (*E*^0^ = −330 to −250 mV vs. NHE) in the active site. The resting state of a dihemic C*c*P is typically a catalytically inactive diferric form, with a His- and Met-coordinated H-heme that participates in ET to the active site, and catalytic bis-His coordinated L-heme that requires activation. It is achieved by reduction of the H-heme, which induces Ca^2+^-dependent spin and coordination change of ferric L-heme to a 5cHS state upon distal Fe_L_-N (His) bond disruption. RR spectroscopy helped characterize these conformational changes on the level of the two hemes that occur in some C*c*Ps in parallel with those rare C*c*Ps that do not require activation [7,8,9]. More recently, RR experiments revealed that some C*c*Ps do not follow either of the abovementioned scenarios, as no significant amount of 5cHS ferric heme population could be detected either in diferric or semi-reduced states. This was rationalized in terms of a novel, more subtle activation mechanism that likely involves formation of a 6c hydroxo complex, which could react with hydrogen peroxide to create the ferric hydroperoxo complex [7,8,9]. 

**Figure 4:**
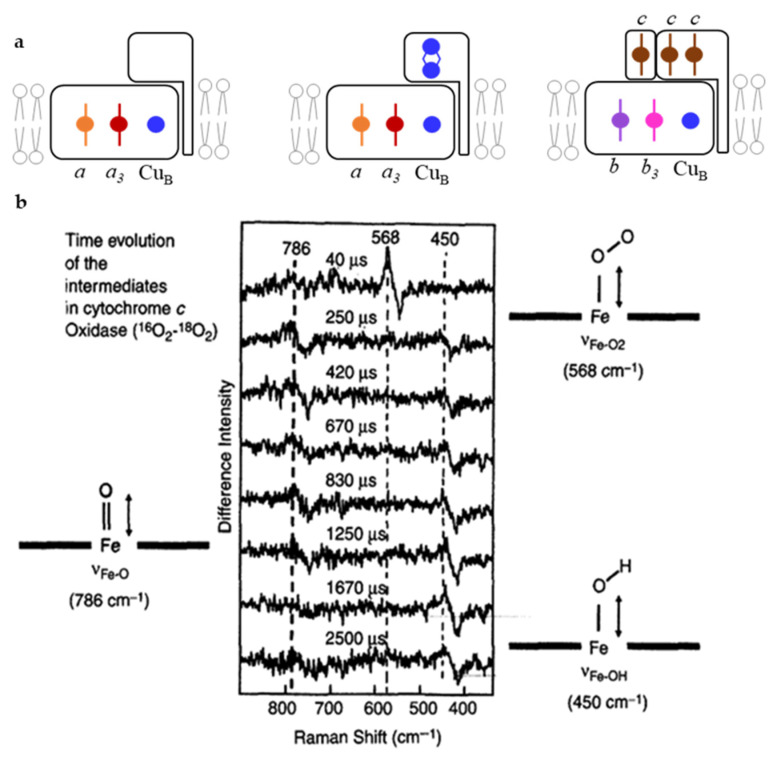
Heme copper oxygen reductases. (**a**) Schematic representation of cofactors in membrane-integrated catalytic (subunit I) and membrane-anchored non-catalytic subunits in *aa_3_* quinol oxidase (QO), mitochondrial *aa*_3_ cytochrome *c* oxidase (C*c*O) and *cbb*_3_-type HCO. (**b**) Low-frequency RR spectra of catalytic intermediates of HCOs. Difference RR spectra of C*c*O reacting with ^18^O_2_ and ^16^O_2_. (**b**) Adapted from Rousseau et al. [57]. Copyright © 2021 Elsevier Science (USA). All rights reserved.

*Oxygen reductase*—Complex IV (cyt *c*: oxygen oxidoreductase, C*c*O, or heme copper oxygen reductase, or heme copper oxidase, HCO) catalyzes the reduction of molecular oxygen to water by utilizing four electrons and four protons. This is one of the most fundamental reactions in living organisms. HCOs pump protons from the N to the P side of the membrane, contributing to the generation of transmembrane electrochemical potential, which drives the ATP synthesis [39]. The catalytic reaction occurs at a binuclear center formed by a HS heme (e.g., heme *a*_3_ in mitochondrial enzyme, here designated as C*c*O) and a copper atom (Cu_B_). The ET to the binuclear center is mediated by a LS heme group in the catalytic subunit (heme *a* in C*c*O) and a copper center in the non-catalytic subunit, which is composed of two copper atoms (Cu_A_) that hold one redox equivalent (Figure 4a middle). The number of non-catalytic subunits and their cofactors vary in HCOs. Bacteria and archaea have complexes that are simpler than the mammalian C*c*O, and if required, the expression of the appropriate HCO (e.g., *aa*_3_-, *cbb*_3_-, or *ba*_3_- type) can be fine-tuned as a function of oxygen pressure levels in the environment. RR spectroscopy played a fundamental role in identification and description of the cofactors in these enzymes, such as (i) the type and spin, oxidation, and coordination state of the heme groups in structurally diverse HCOs of different origin [40,41,42,43,44] and (ii) interactions, conformations, and the dynamics of small ligand (CO, CN^−^, N_3_, NO) binding [45,46,47,48,49,50,51]. 

The low-frequency region of RR spectra has provided unprecedented details on (i) mechanistic properties of HCOs, including detection and identification of the short living catalytic intermediates formed upon oxygen binding to the catalytic HS heme (i.e., *a*_3_, *b*_3,_ *o*_3_) and (ii) molecular environment and conformation of the catalytic site, based on correlation of Fe-CO and Fe-C-O stretching mode frequencies of HS heme-CO adduct [52,53]. Moreover, protonation events during the catalytic reaction and in particular the role of the two HS heme propionates and the highly conserved amino acid residues found in their vicinity, have been elucidated by RR spectroscopy using structurally different HCOs [50,54], isotopic substitution, wavelength selective resonance enhancement, and mutagenesis studies [55]. A presence of *a*-type hemes in some HCOs offers further advantages as the effect of mutations and other molecular perturbations can be monitored via porphyrin formyl stretching C=O modes, which are well resolved in RR spectra of ferrous HS and LS hemes at 1661 and 1628 cm^−1^, respectively. In this manner, the frequencies of *a* (C=O) and *a*_3_ (C=O) stretching modes reveal the respective heme environments [56]. They highlight the importance of H bonding interactions stabilizing the heme *a* and hydrophobic environment surrounding the heme *a*_3_ in an *aa*_3_-type HCO and the role of the specific amino acids that participate and ensure a proper environment and H network [54]. 

Perhaps the most significant contribution of RR spectroscopy of heme proteins can be attributed to the understanding of the complex catalytic reaction of HCOs, as it provided identification and structural characterization of the transient intermediate species and kinetics of their formation. Reduced HCOs bind molecular oxygen and reduce it via formation of short living A, P, F, and H intermediates [57], which were initially only tentatively described by electronic absorption spectroscopy. RR experiments performed independently by the groups of Kitagawa, Rousseau, and Babcock led to a consistent mechanistic model of HCOs, employing isotopic labeling, mutagenesis studies, a number of structurally different HCOs, and individually developed TR RR experimental approaches [39,57,58,59,60,61]. In particular, iron–oxygen stretching frequencies obtained by TR RR ^16^O_2_–^18^O_2_ difference spectra, measured in H_2_O and D_2_O media, helped identify proton-coupled ET reactions and establish structural fingerprints of each catalytic intermediate (Figure 4b). These include the first formed compound A, assigned to ferric-superoxide species with iron–oxygen stretching frequency at 568 cm^−1^, compound P (iron–oxygen stretching frequency at 804 cm^−1^, electronic transition at 607 nm), and compound F (iron–oxygen stretching frequency at 786 cm^−1^, electronic transition at 580 nm). The P and F are both attributed to oxoferryl, Fe^4+^ = O, species with subtle differences in the proximal His ligand of *a*_3_ and one of its propionates, and/or the presence of a nearby amino acid (Tyr) radical [57,62]. The intermediate F decays into hydroxyl H species, with Fe-OH stretching frequency of 450 cm^−1^, Figure 4b.

IR difference spectroscopy recorded under steady state conditions and in the TR mode identified protonation/deprotonation and re-protonation events during the catalytic cycle of *aa*_3_-type HCO. The data in particular point out the role of a proton shuttle of glutamic acid found at ~11 Å from the active site, which changes its protonation state a number of times during the catalytic cycle. The role of the Tyr located in the proximity of the binuclear center in the splitting of the O-O bond during the catalysis was also highlighted by IR spectroscopy [63].

**Figure 5:**
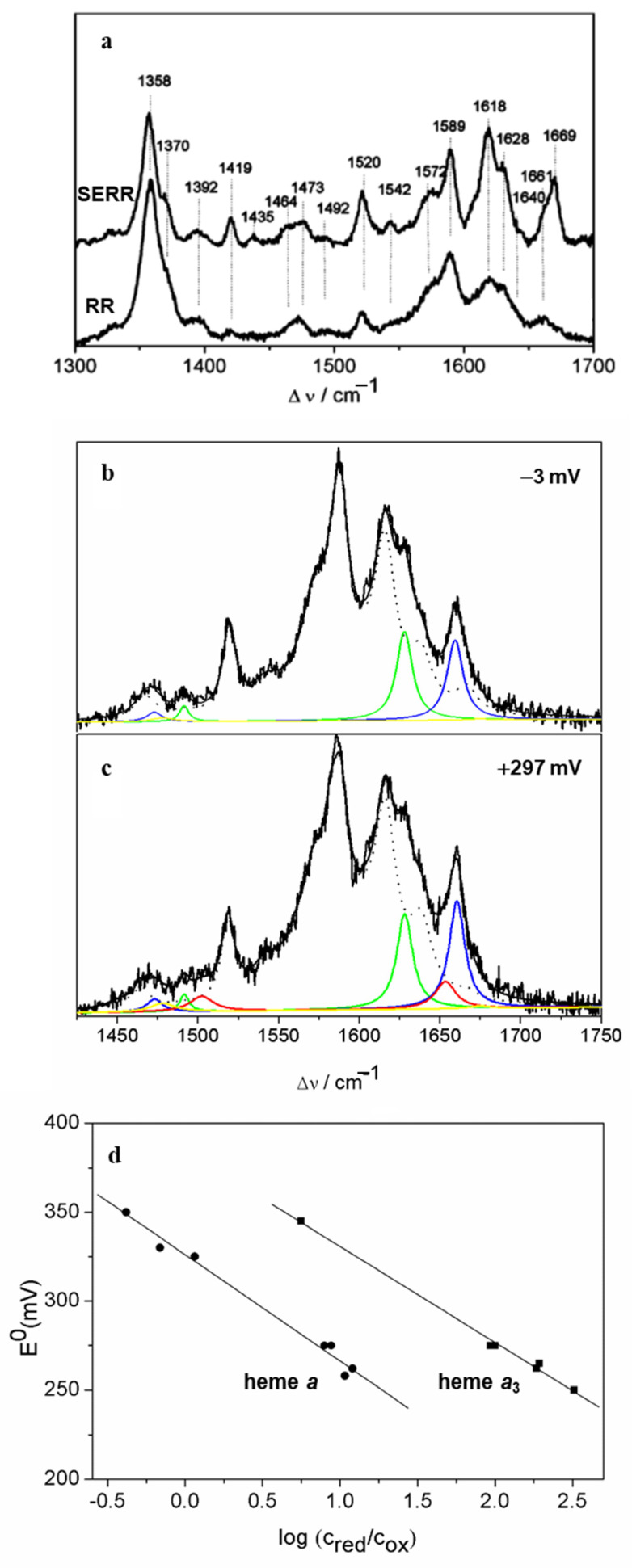
*aa*_3_ quinol oxidase (QO). (**a**) SERR spectrum of ferrous QO immobilized on Ag electrode at a potential of −403 mV vs. NHE and RR spectrum of QO in solution reduced with dithionite. (**b**,**c**) SERR spectra of the immobilized QO at electrode poised potential of (**b**) −3 and (**c**) +297 mV vs. NHE; component spectra represent the overall simulated spectra (black), ferrous heme *a* (green), ferric heme *a* (yellow), ferrous heme *a*_3_ (blue), ferric heme *a*_3_ (red), and the envelope containing all other bands common to hemes *a* and *a*_3_ (dotted black). (**d**) Nernst plots for the redox processes of immobilized QO. Adapted from Todorovic et al. [16]. Copyright © (2005) American Chemical Society.

More recently, SERR spectroscopy provided a novel platform for investigations of HCOs under conditions that can mimic some basic features of their natural environment. Since these enzymes exert their function integrated into a phospholipid bilayer under restricted mobility, directionalized ET from electron donor to LS heme and binuclear site, and influence of strong interfacial electric fields, the immobilization onto biocompatible electrodes mimics far better these conditions than the solution studies [16]. Furthermore, SERR potentiometric titrations represent a powerful alternative to common methods for the determination of the *E*^0^ value, which is a prerequisite for understanding the ET pathway in these enzymes. The determination of *E*^0^ of the individual heme groups of HCOs by conventional electronic absorption titrations is hampered by strong overlapping of the spectra of individual hemes and by complex cooperative effects that modulate the electroprotonic energy transduction [16]. To that end, an *aa*_3_-type quinol oxidase (*aa*_3_ QO) that contains the catalytic and one cofactor-free non-catalytic subunit (Figure 4a) was attached to detergent-coated Ag electrodes by spontaneous adsorption. A comparison of RR and SERR spectra of the enzyme in solution and adsorbed states revealed that the enzyme preserved its native structure upon immobilization (Figure 5a). Deconvolution of the (SE)RR spectra by component analysis (Figure 5b,c) allowed separation of the contributions from the LS and the HS hemes based on their respective ν_3_ and ν_C=O_ modes, among more than 40 vibrational modes originating from the two hemes [16]. The potential dependence of these spin- and redox-state-sensitive marker bands allowed for determination of midpoint redox potentials of hemes *a* (*E*^0^ = 320 mV vs. NHE) and *a*_3_ (*E*^0^ = 390 mV vs. NHE), which reveal a reversed order of reduction compared to mitochondrial-like HCOs. This suggests a distinct mechanism of electroprotonic energy transduction in *aa*_3_ QO in comparison with, e.g., C*c*O (Figure 5d). A downhill ET is already guaranteed by the order of the midpoint redox potentials at the onset of enzyme reduction, indicating that this enzyme does not require a complex network of cooperativities to ensure exergonicity. Another SERR study employed a more complex, pentahemic *cbb*_3_-type HCO, anchored to nanostructured Ni-NTA-coated Ag electrodes and further embedded into a lipid bilayer that mimics the natural membrane, which was catalytically active within the electrode construct [64]. This strategy, which allows the control of the enzyme orientation that can be probed by SEIRA spectroscopy, was first developed in the studies of *aa*_3_-type HCO [17,65,66]. SERR potentiometric titrations of the whole *cbb*_3_-type HCO complex and of the two individually expressed non-catalytic subunits, indicate that the dihemic subunit can be considered redundant for ET and catalysis, supporting the hypothesis that it plays a role in oxygen sensing. SERR also helped clarify the poorly understood coupling between heme reduction and proton translocation in HCOs and in particular the involvement of heme *a*_3_ propionates in the proton pumping. The analysis was based on the protonation dependent CH_2_ propionate bending modes that have been detected by H_2_O-D_2_O (SE)RR difference spectroscopy. This allowed individual assignment of all four heme propionates. The data support the hypothesis that heme *a*_3_ propionates act as possible proton loading sites in HCOs [67].

IR difference spectroscopy was employed to specifically probe the heme propionate protonation events using a C*c*O mutant with ^13^C labeled propionates to distinguish its signal from possible concomitant changes of CO stretching modes of carboxylic acid groups of side chains [18]. In such a way, differential IR spectra, employing isotopically labeled ligands or different redox states of the protein (e.g., oxidized minus reduced), provided further structural details on active site conformations in HCOs and specific protonatable sites [68,69]. It was demonstrated that NO binding to *cbb*_3_, which also efficiently reduces NO to N_2_O, exclusively occurs via HS heme *b_3_*, and not via Cu_B_ as suggested [70]. Step-scan IR spectroscopy, using Cu_B_-CO adduct as a probe, showed that the protonation events in the binuclear site of *ba*_3_-type HCO involve Tyr residues [71]. Moreover, electrochemical redox titrations of *aa*_3_- and *caa*_3_-type HCOs, performed using IR spectroscopy in the transmission and the ATR mode, helped identify the redox-sensitive bands originating from heme groups, their ligands, amino acid residues and/or protein backbone. This strategy allowed for tentative assignment of the redox transitions of the individual redox centers [36,68,72]. A similar approach, in which electrochemistry coupled to redox induced IR difference spectroscopy was used to address the *E*^0^ of the heme *b*_3_ in *cbb*_3_ HCOs, revealed relatively low midpoint redox potential of this HS heme. It was rationalized in terms of a unique coordination scheme involving hydrogen bonding between the His ligand of the heme *b*_3_ and a highly conserved glutamic acid, which participate in coupled electron and proton transfer steps and facilitate ET between LS and HS hemes during turnover [73].

*Nitric oxide reductase* (NOR) catalyzes the two-electron reduction of NO to N_2_O at the di-nuclear heme *b*_3_-Fe_B_ center. Similarly to the active site of HCOs, the heme *b*_3_ is in the HS state, while Fe_B_ is a non-hemic iron atom. NORs in addition house *c*-type and *b*-type LS hemes. RR spectroscopy provided evidence about NO reduction by monitoring the events that occur upon NO binding to the catalytic *b*_3_-type heme. Specifically, formation of the N-N bond and the fate of the proximal His-heme *b*_3_ bond along this process, as well as the recovery of the *b*_3_-O-Fe_B_ state, have been disentangled by RR spectroscopy [74].

## 3. Fe-S Proteins

### 3.1. Hydrogenases

**Figure 6:**
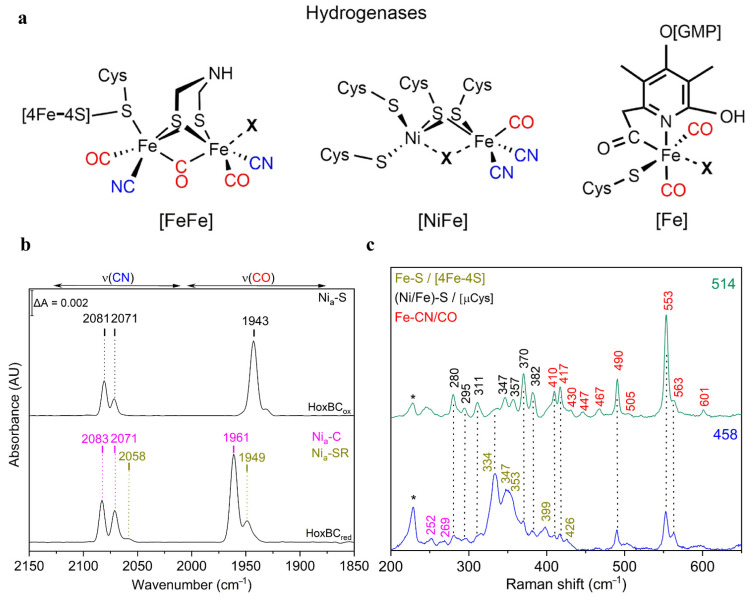
(**a**) Schematic representation of the active sites of [NiFe], [FeFe] and [Fe] hydrogenase. In [NiFe] hydrogenases, the nickel ion is coordinated by four cysteine residues, two of which are shared with the iron ion. One CO and CN^−^ ligands complete the coordination sphere of the iron. In [FeFe] hydrogenases, the catalytic center is the H-cluster comprising a di-iron (2Fe_H_) and a [4Fe-4S]_H_ site sharing a cysteine coordination. Each iron of the 2Fe_H_ is coordinated by one CN^−^ and one CO ligand. A bridging CO and an azadithiolate ligand complete the 2Fe_H_ coordination. In [Fe] hydrogenases, the single iron is coordinated by two CO ligands, a cysteine thiolate, and a guanylyl pyridinol cofactor bound to the iron via its acyl-carbon and pyridinol–nitrogen. Substrates and inhibitors bind at the coordination site marked by X. (**b**) IR spectra of as-isolated (top trace, Ni_a_-S intermediate) and H_2_-reduced (bottom, Ni_a_-C and Ni_a_-SR intermediates) RH (HoxBC) at 283 K displaying the ligand stretching vibrations of the CO and CN^−^ ligands of the active site, located between 1890 and 2000 cm^−1^ and 2030 and 2120 cm^−1^, respectively. (**c**) Low-temperature RR spectra at 80 K of the regulatory [NiFe] hydrogenase (RH) recorded with excitation at 458 (blue trace) and 514 (green trace) nm in its as-isolated form. Color code of various metal ligand vibrations: Fe-CN/CO (red); Ni/Fe-S of bridging cysteines (black); Fe-S of [4Fe-4S] clusters (dark yellow). The lattice modes of ice are denoted by an asterisk (*).

Hydrogenases catalyze the reversible splitting of molecular dihydrogen (H_2_) into protons and electrons (or hydride species), often with high catalytic rates, and can be classified into [NiFe], [FeFe], and [Fe]-hydrogenases, depending on the particular metal composition of their catalytic centers [75]. These metalloenzymes have been extensively studied in recent years, revealing new insights into the mechanistic details of the H_2_ splitting, which are regarded as benchmarks for the development of biomimetic and bioinspired catalysts for H_2_ production and/or uptake [76]. In this context, vibrational spectroscopy has greatly contributed to the understanding of hydrogenases, targeting inorganic cofactors, such as the particular catalytic site and Fe-S cluster relays, as well as organic protein cofactors (e.g., FAD and FMN) and certain adjacent protein residues (e.g., cysteine, glutamate) all involved in the biological H_2_ conversion [77]. Thereby, IR spectroscopy has played a major role in the characterization of these enzymes, which is related to the presence of the unusual inorganic carbon monoxide (CO) and cyanide (CN^−^) ligands coordinated to the particular active sites (Figure 6a) that exhibit characteristic IR bands in a spectral region free of other protein absorptions. Noteworthily, the specific band position of the CO (1780–2030 cm^−1^) and CN^−^ (2030–2150 cm^−1^) stretching modes (Figure 6b, Table 1) are particularly sensitive to changes of the local protein environment, oxidation states of the metal centers, as well as external perturbations induced by changes of e.g., temperature and pH [78,79]. Solution IR spectro-electrochemical studies [80] were thus extremely helpful for the structural and electronic characterization of the different redox states, providing key insights into the catalytic mechanism. Briefly, the [NiFe] hydrogenase catalytic cycle comprises four intermediates, i.e., Ni_a_-S, Ni_a_-L, Ni_a_-C, and Ni_a_-SR differing in the oxidation state of nickel (Ni^1+/2+/3+^) while iron retains a Fe^2+^ LS configuration throughout the entire catalytic cycle. In the [FeFe] hydrogenase catalytic cycle, the di-iron center, 2Fe_H_, has been isolated in a variety of intermediates, some of which are well understood (H_ox_, H_red_/H_red_H^+^, H_hyd_) while other presumed intermediates (H_red_’H, H_sred_, H_hyd_H^+^, H_ox_H^+^) are still a subject of intense discussion [81,82]. For understanding the assignment of the catalytic competency of hydrogenase intermediates, a number of studies employing ATR-IR [22] and SEIRA [20] spectroscopy, both under controlled gas atmosphere or/and potential, photogating with time-resolved IR spectroscopy [83], and protein film IR electrochemistry (PFIRE) [21] have been performed.

Recently, ultrafast pump-probe and two-dimensional (2D) IR provided novel insights into the dynamic interactions between the hydrogenase CO/CN^−^ ligands and the complex interplay between the [NiFe] site and the protein scaffold [84]. Apart from the distinct CO and CN^−^ markers bands, IR spectroscopy has also unveiled the participation of protein residues in the stabilization of certain intermediates. Combined IR difference spectroscopy and hydrogen/deuterium (H/D) isotopic labeling allowed for direct monitoring of the changes in the protonation state of conserved glutamate and arginine residues in both [NiFe] and [FeFe] hydrogenases [85]. Noteworthily, the IR detection of S-H/S-D stretching bands at 2505 cm^−1^ and 1822 cm^−1^, respectively, confirmed the protonation of a Ni-bound cysteine in the Ni_a_-L intermediate.

Additional understanding of mechanistic properties of hydrogenases has been derived from RR spectroscopy that enables the detection of multiple protein cofactors. RR spectra of the F-420 [NiFe] hydrogenase revealed the spectral contributions of FAD, Fe-S clusters, and the [NiFe] active site [86]. Notably, 458 nm laser excitation preferentially enhanced the metal-ligand modes of the Fe-S clusters (Figure 6c, blue trace, Table 1), while the excitation lines distant from the S → Fe CT transition resulted in a preferential enhancement of the active site vibrational modes (Figure 6c, green trace, Table 1) [87,88]. Supported by theoretical methods, RR spectroscopy has shown to be highly versatile in unraveling key electronic and structural details of the [NiFe] and [FeFe] bimetallic centers. Additionally, RR and IR spectroscopic studies were successfully performed on the same single hydrogenase crystals, complementing the crystallographic data and facilitating structural assignment of ambiguous inhibited and active redox states, such as the oxygen-stable H_inact_ and the hydrogen-binding intermediate Ni_a_-S state in [FeFe] and [NiFe] hydrogenases, respectively [86,89]. Notably, well-defined redox states in hydrogenase single crystals for IR imaging have been generated by electrochemical and gas control [90,91,92,93]. Furthermore, Fe-OH ligation at the unprecedented [4Fe–3S] cluster of the O_2_-tolerant membrane-bound [NiFe] hydrogenase from *R. eutropha* has been identified by RR spectroscopy and confirmed by theoretical quantum and molecular mechanics methods [94,95]. The corresponding FeS region of the three different clusters in the enzyme has been disentangled recently by RR spectroscopy in combination with protein engineering and X-ray crystallography [96], while its coupling with the physiological electron acceptor, cytochrome *b*_3_, was investigated by SERR spectro-electrochemistry [21,97].

### 3.2. Multi-Cluster Containing Ferredoxins

*Ferredoxin,* Fd, is a small ET protein that can have one or more Fe-S clusters and that takes part in a number of metabolic reactions. Multiple wavelength excitation of Fe-S proteins that contain different clusters can assure selective RR enhancement of individual clusters and provide their independent monitoring. IR and RR spectroscopies of a Fd that houses one [3Fe-4S]^1+/0^ and one [4Fe-4S]^2+/1+^ center have addressed the effect of thermal perturbation on the level of secondary structural elements and the individual clusters, respectively. RR spectroscopy provided fine details about the disassembly of the centers in Fd, on the level of bridging and terminal Fe-S bonds of each cluster [98]. Three different laser excitation lines (413, 458, and 514 nm) were used to achieve a differential enhancement of the RR bands associated with [3Fe-4S] and [4Fe-4S] centers. For all three excitation wavelengths, the most prominent RR band is observed at 346 cm^−1^, which is a fingerprint of the [3Fe-4S] cluster bridging mode, [3Fe-4S^B^]. Terminal vibrations of the same cluster, [3Fe-4S^T^], are found at 366 cm^−1^. The RR spectra recorded with 413 nm and 458 nm excitation show two additional features at 336 cm^−1^ and 358 cm^−1^ that are indicative of bridging and terminal modes of the [4Fe-4S] cluster, respectively ([4Fe-4S^B^] and [4Fe-4S^T^]). The spectral contributions of the clusters were accurately determined by applying band-fitting procedures. The intensity changes of the individual RR bands, i.e., [3Fe-4S^B,T^] and [4Fe-4S^B,T^], upon successive incubations of Fd at increasing temperatures revealed an apparent transition at around 53 °C for all vibrational modes. The simultaneous degradation of the two metal centers follows the initial loss of the alfa helical structure and triggers major structural changes of the protein matrix, including the loss of tertiary contacts and a secondary structure reorganization that is consistent with the formation of a molten globule state, which could be assessed by IR spectroscopy [98].

## 4. Outlook

The continuous progress in experimental methodologies that ensure improved metalloprotein expression systems and purification and selective isotopic labeling methods, alongside the development of faster, more sensitive, and sophisticated vibrational spectroscopic techniques, allow us nowadays to study exceptionally complex, dynamic, and transient metal centers and processes in which they participate. For example, in the past few years, the synchrotron Mössbauer technique, nuclear resonance vibrational spectroscopy, NRVS, has greatly contributed to the understanding of iron dynamics in certain hydrogenase intermediates. Considerable achievements in particular resulted from the development of biochemical and chemical procedures enabling selective ^57^Fe-labeling exclusively at the [NiFe] and [FeFe] bimetallic centers [99,100]. Similarly, recent advances in femtosecond-stimulated Raman spectroscopy (FSRS), and in particular, the unique power of tunable FSRS to achieve different resonance conditions (i.e., FSRRS) and track various species and states of heme proteins, allow nowadays studies of protein dynamics and energy flow with femtosecond resolution [101,102]. To that end, well established RR, SERR, IR, and SEIRA spectroscopies, complemented with these more recently developed methods and theoretical calculations, have an unforeseen future in discovering new roles and physiologically relevant forms, metal centers with unique structures, and the processes that occur in multiple cofactor-containing metalloenzymes.

## Figures and Tables

**Table 1:** Assignment of characteristic resonance Raman (RR) and IR active vibrational modes found in metalloprotein. Heme proteins―typical frequency range of the RR marker bands (*ν_i_*) of ferric and ferrous His/Met and His/- coordinated *c*-type heme. Iron-sulfur proteins―typical frequency range of predominantly bridging (B) and terminal (T) Fe-S RR modes for different cluster types. Hydrogenases―typical frequency regions of IR and RR bands for metal bound CO and CN^−^ ligand vibrations.

		Heme Group (1300–1700 cm^−1^)				
Coordination and Spin State		Redox State	*ν*_4_	*ν*_3_	*ν*_2_	*ν*_10_
His/Met	6cLS	3+ 2+	1374 ± 2 1363 ± 2	1505 ± 3 1495 ± 3	1586 ± 2 1592 ± 3	1638 ± 3 1624 ± 3
His/-	5cHS	3+ 2+	1371 ± 2 1357 ± 3	1496 ± 2 1472 ± 1	1576 ± 2 1573 ± 3	1627 ± 4 1606 ± 2
		**Fe-S Clusters (200–450 cm^−1^)**				
**Cluster**	**RR Active Redox State**		**Frequency Range (cm^−1^)**			
1Fe-4S	3+		314–318			
2Fe-2S	2+		281–290 (T)			
3Fe-4S	1+		346–348 (B)			
4Fe-4S	2+/3+		333–339 (B)/341–344 (B)			
		**[NiFe] and [FeFe] Hydrogenase Active Sites**				
**Hydrogenase**	**Bands**		**Frequency Range (cm^−1^)**			
NiFe/FeFe	ν(CO)	IR	1780–2030			
NiFe/FeFe	ν(CN)	IR	2030–2150			
NiFe/FeFe	Fe-CN/CO	RR	400–650			
FeFe	Fe-S [adt]	RR	300–400			
NiFe	(Ni/Fe)-S [µCys]	RR	280–400			
NiFe	Fe-OH [4Fe-3S]	RR	500–600			

## Data Availability

Not applicable.
